# Practice reduces the costs of producing head fakes in basketball

**DOI:** 10.1007/s00426-023-01885-x

**Published:** 2023-10-13

**Authors:** Nils Tobias Böer, Matthias Weigelt, Christoph Schütz, Iris Güldenpenning

**Affiliations:** 1 Department of Sport & Health, Paderborn University, Warburger Str. 100, 33098 Paderborn, Germany; 2 Faculty of Psychology and Sports Science, Bielefeld University, Universitätsstraße 25, 33615 Bielefeld, Germany

## Abstract

Previous research indicates that performing passes with a head fake in basketball leads to increased response initiation times and errors as compared to performing a pass without a head fake. These so-called fake production costs only occurred when not given the time to mentally prepare the deceptive movement. In the current study, we investigated if extensive practice could reduce the cognitive costs of producing a pass with head fake. Twenty-four basketball novices participated in an experiment on five consecutive days. A visual cue prompted participants to play a pass with or without a head fake either to the left or right side. The cued action had to be executed after an interstimulus interval (ISI) of either 0 ms, 400 ms, 800 ms or 1200 ms, allowing for different movement preparation times. Results indicated higher response initiation times (ITs) and error rates (ERs) for passes with head fakes for the short preparation intervals (ISI 0 ms and 400 ms) on the first day but no difference for the longer preparation intervals (ISI 800 ms and 1200 ms). After only one day of practice, participants showed reduced fake production costs (for ISI 0 ms) and were even able to eliminate these cognitive costs when given time to mentally prepare the movement (for ISI 400 ms). Accordingly, physical practice can reduce the cognitive costs associated with head-fake generation. This finding is discussed against the background of the strengthening of stimulus response associations.

## Introduction

Interactions between athletes are an essential part of many competitive sports. In such interactions, fakes are often used to deceive the opponent about one’s own intentions and to gain an advantage for the genuine action (Güldenpenning et al., 2017; Jackson & Cañal-Bruland, 2019). For example, a basketball player, who wants to pass the ball to a teammate at the right side, turns his head to the opposite side a little before initiating the passing action (so-called head fake; Polzien et al., 2021). The deceptive head movement interferes with the processing of the pass direction and, therefore, makes it difficult to recognize the “true” action intention of the player performing the head fake (Kunde et al., 2011). Research on fake actions in sports is growing in the last years, showing their effectiveness in competitive settings (e.g., 73% of basketball shot fakes in the NBA are successful and advantageous for the attacker; Meyer et al., 2022a). While in previous years most research focused on investigating the efficiency and boundary conditions of fake actions on the side of the observer (cf. Güldenpenning et al., 2017 for a review), costs of fake actions, which occur on the side of the performer (i.e., fake production costs), have only been sparely investigated (Güldenpenning et al., 2023; Kunde et al., 2019; Wood et al., 2017). The present study aims to further contribute to the understanding of fake production costs using the example of head fakes in basketball. Specifically, the study investigates how physical practice influences the fake production costs among basketball novices.

Passes with head fakes in basketball have been shown to increase reaction times (RT) and error rates (ER) of the defending opponents compared to passes without head fakes (Kunde et al., 2011). This head-fake effect and factors which might modulate its size have already been extensively investigated in recent years, for example, the proportion of fake trials (Alhaj Ahmad Alaboud et al., 2012; Güldenpenning et al., 2018), the role of practice with the task (Güldenpenning et al., 2020b), the role of motor and visual training and basketball expertise (Güldenpenning et al., 2022), different avoidance instructions (Güldenpenning et al., 2019), cognitive load (Güldenpenning et al., 2020a), and some others (Friehs et al., 2019; Güldenpenning et al., 2020c; Güldenpenning et al., 2022; Polzien et al., 2020; Weigelt et al., 2017, 2020). All studies point out that the head-fake effect is robust against a number of factors, that is, it persists in all conditions and manipulations to a significant degree. Do these results mean that the basketball player performing a head fake gains an unrestricted advantage through the deception? Or can the execution of a head fake itself also result in disadvantages on the side of the deceiver?

In a recent study, Güldenpenning et al. (2023) assessed fake production costs of generating head fakes in basketball. They expected to find motor programming costs for a pass with a head fake as compared to a pass without a head fake. This could be caused by response-response incompatibility costs (Hazeltine, 2005; Heuer, 1995; Peterson, 1965) due to the generation of two spatially incompatible body movements (e.g., head turn to the left, passing the ball to the right). At least for bimanual actions, response-response incompatibility costs are evident in increased reaction times, movement times (MT), and lowered accuracy of two mutually incompatible actions (e.g., moving both fingers simultaneously, one vertically and one horizontally) compared to compatible actions (e.g., moving both fingers simultaneously in the same direction with the same trajectory; Hazeltine et al., 2003). These costs can be reduced when participants were given enough time to prepare the movement (e.g., Stimulus Onset Asynchrony (SOA) of 1 s delay between two Stimuli presented in a trial; see Spijkers et al., 1997). To evaluate whether response-response incompatibility costs, which arise in the process of response selection (Hazeltine et al., 2003), could be the source of the fake production costs, Güldenpenning et al., (2023) tested different inter stimulus intervals (ISIs) to mentally prepare the production of a pass with a head fake or without a head fake. The reasoning was as follows: An increase of the length of the ISIs should reduce or even eliminate potential fake production costs (i.e., the difference in initiation times (IT), MT, and ER between passes with and without head fake), as the response selection process should have been completed beforehand (Wirth et al., 2016).

Güldenpenning et al. (2023) conducted two, slightly different, cued-choice reaction tasks. In Experiment 1, auditory cues (440 Hz or 1200 Hz sinus or jigsaw wave) were used to determine if the novice participants had to perform a pass with or without a head fake, either to the left or to the right side. In Experiment 2, these response movements were cued by a visual stimulus of a defending basketball player (either red or blue t-shirt, covering either the left or right side) to better mimic a realistic situation from basketball. Both experiments revealed higher ITs for passes with head fakes compared to passes without head fakes for the short to medium length ISI (from ISI 0 ms to ISI 800 ms), while there were no differences for the longer preparation intervals (ISI 1200 ms and 1500 ms). These results clearly show that performing a head fake comes with costs, which can be overcome if the deceiving person has time to mentally prepare the action. The fake production costs, which are derived from the difference between passes with and without a head fakes, found in this study cannot be explained solely by the complexity of the external stimulus as it was identical for both passes with and without head fakes. Since these costs show the typical course with a decrease for longer preparation intervals, fake production costs for the head fake seem to be caused by response-response incompatibility effects.

Another recent study by Kunde et al., (2019) investigated the cognitive costs associated with the generation of a one-handed fake throw in an interaction scenario in which two people threw or faked to throw a hacky sack ball into a small target-basket at the side of their opponent, respectively. More specifically, participants took part in pairs and were assigned in turns to the role of the attacking or defending player. While the attacking player was either instructed to throw or fake to throw the ball, the defending player either had to intercept the throw before it hit the basket or to inhibit the response. The results showed higher response initiation times for the production of fake throws compared to non-fake throws, indicating fake production costs. Interestingly, longer response initiation times seemed to be an indicator for the defender that a fake action will be performed. A prolonged initiation time of 100 ms increased the chance that a defender classified the action of the attacker as fake by more than 10% (Kunde et al., 2019). From a practical point of view, it is therefore of particular relevance for athletes if such fake production costs can be overcome, as an opponent might be more likely to expect a deceptive action if the action initiation time increases. Thus, for the deception to be maximally successful, the attacking player must minimize the time costs for action initiation. Accordingly, we studied if the extensive practice (i.e., practice over five consecutive days) of passes with and without head-fakes is sufficient to decrease fake-production costs in basketball. The aim of this study was to further contribute to the understanding of fake production costs of head fakes in basketball in novices and whether these costs can be modulated by practice.

As argued above, fake production costs seem to occur during response selection. Response selection can, under some circumstances, be automatic, meaning that it can be transferred to the stimulus (Jong et al., 1994). Extensive practice might strengthen stimulus–response associations, such as those used in our experiment, and lead to conditional automaticity (Hommel, 2000). Specifically, an automatic translation of a stimulus into a response implies that the action selection process, which is the origin of the production costs of a head fake (Güldenpenning et al., 2023), can be skipped because the response is already uniquely specified by the stimulus. Accordingly, it can be assumed that the production costs of a head fake will be reduced or even eliminated after extensive practice.

To test this assumption, the previously described design from the second experiment of Güldenpenning et al. (2023) was used and the number of trials were expanded from 240 to 1600 trials (i.e., 320 trials each day over 5 consecutive days). Similar to Güldenpenning et al. (2023), increased response initiation times (ITs) and error rates (ERs) are expected for the production of passes with head fakes compared to passes without head fakes on the first day of practice, i.e., signifying head-fake production costs. These costs should decrease (or even vanish), when the required action is cued in advance, and thus, action selection processes are removed from the initiation time interval. Also, head-fake production costs should decrease (or even vanish) with increasing practice (from Day 1 to Day 5).

Additionally, the coefficient of variation of the IT (IT_cv_) was analyzed, which is calculated by dividing the standard deviation of the chosen reaction time parameter (here: initiation time) by the mean of the IT of one individual, multiplied by 100 (Guildford, 1956). The coefficient of variation is a relative variability measure, calculated for each participant and day of practice. Previous studies could show that the IT_cv_ is indifferent to effects of repeated testing (Flehmig et al., 2007). Therefore, a reduction of the IT_CV_ independent of the factors *ISI* and *day of practice*, together with a general reduction of the ITs of the participants, would be an indicator that the performance increase of the participants (as signified by smaller fake production costs) is not caused by simple test repetition effects but may be related to effects of practice. We expected that the IT_cv_ would decrease with increasing practice (from Day 1 to Day 5), both for passes with and without head fakes.

Taken together the following predictions were made: if response-response incompatibility costs are the source of the fake production costs, the initiation time and error rate should be higher when participants have to perform a pass with head fake with no or short ISI (i.e., 0 ms, 400 ms, 800 ms) on the first day of physical practice, while there should be no differences when the participants have enough time to mentally prepare the required answer (e. g., ISI of 1200 ms). Also, the fake production costs (measured by IT and ER) and the IT_cv_ should be reduced with increasing practice from Day 1 to Day 5.

## Methods

### Participants

The previous study of Güldenpenning et al., (2023) suggested a large effect size for the production costs of the head fake in dependency of the ISI, and the sample was planned accordingly. Specifically, for an interaction effect between *type of pass*, *ISI*, and *day of practice* of *f* = 0.50, a power of 1−*β* = 0.90, and an α-value of 0.05, a sample size of at least 24 participants was planned. Calculations were carried out using G.Power 3.1.9.7 (Faul et al., 2007).

Twenty-five sports science students at the Paderborn University were tested, but one participant was excluded from data analysis as data was missing from the first measurement due to technical problems. The remaining twenty-four volunteers (5 females, mean age = 24.6 years, *SD* = 2.4) participated in the experiment without payment but received course credit. None of them had basketball experience beyond leisure sports activities. All participants reported normal or corrected-to-normal vision and all of them had no knowledge of the expected outcome of this experiment. The participants were not screened for impaired hearing but were asked after the test block on the first day whether they had problems seeing the stimulus material or hearing the auditive Go-signal.

The study was conducted in accordance with the German Psychological Society (DGPs) ethical guidelines (2004, CIII). This research was also reviewed by the ethics committee of the Paderborn University. All procedures performed in the study were in accordance with the 1964 Helsinki declaration and its later amendments. Participants provided written informed consent that their data will be anonymously (i.e., without access to their names) saved, analyzed, and published.

### Apparatus, stimuli and procedure

**Fig. 1:**
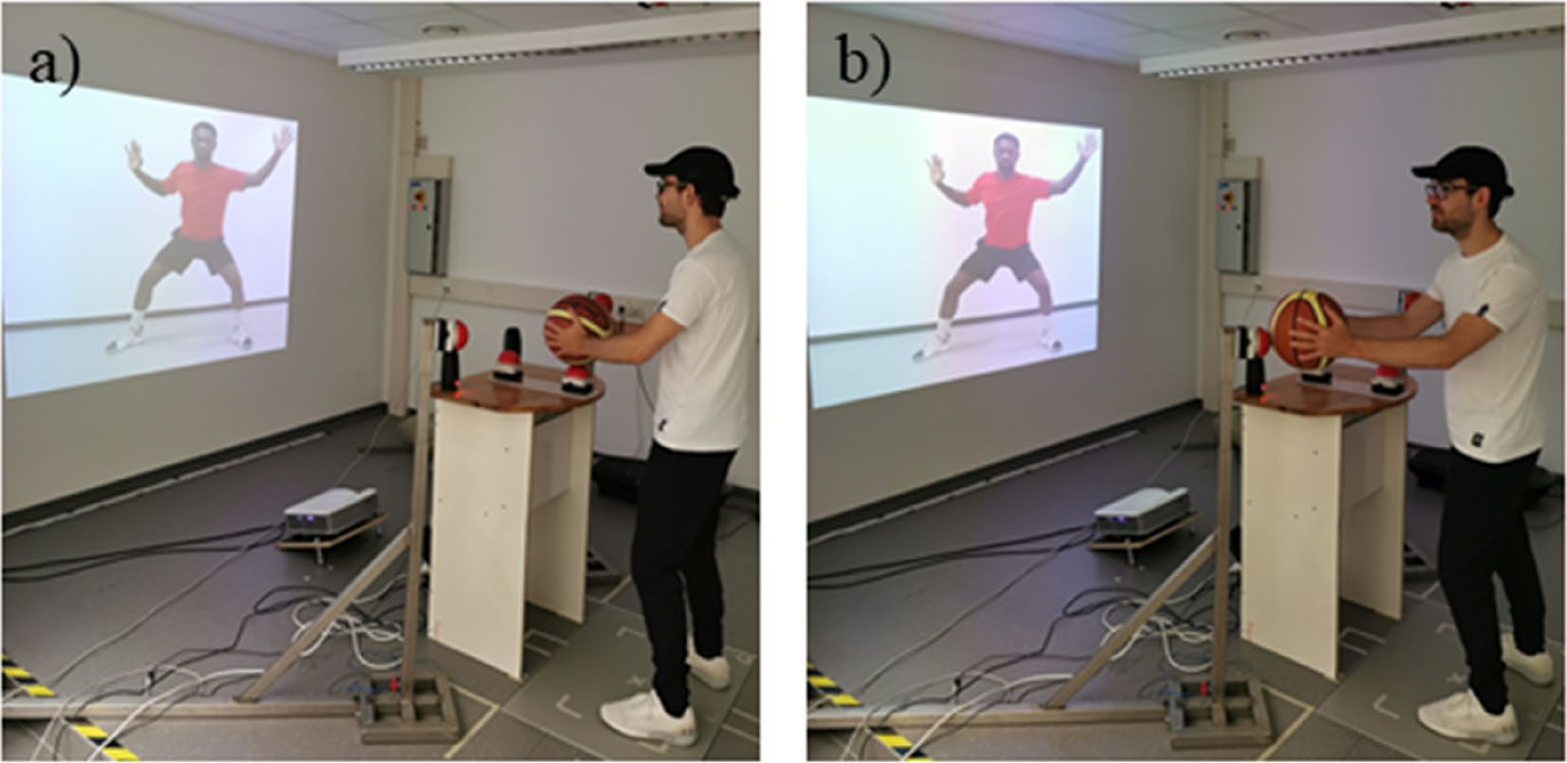
Setup of the Experiment. Setup of the experiment, exemplarily shown for a visual stimulus of a basketball player who covers the right side (from the perspective of the observer). Therefore, the participant has to imitate a passing movement to the left side. Here, the red jersey signals that a passing movement without head fake has to be performed. Picture **a** shows the participant with the basketball on the starting position waiting for the auditory GO signal. Picture **b** shows the end of the pass without head fake to the left side with the participant pressing the left button with the basketball

The experimental setup is identical to our previous study (Güldenpenning et al., 2023). The participants were placed at a distance of 250 cm in front of a screen wall, standing at an apparatus on which basketball passes with or without a head fake could be executed and which allowed to measure ITs, ERs, and MTs. This apparatus consisted of two custom-made steel holdings, which were placed to the left and to the right of a desk, with mounted buttons (height: 1.20 m; distance between buttons: 1.25 m) for the participants to press on with the basketball to indicate a pass to the left or to the right with one button placed between them on a desk in front of the participants (height: 1 m; cf. Figure 1).

The static stimulus material consisted of two different basketball players, one wearing a red shirt and the other a blue one. The images showed the basketball players performing a defensive movement to one side by blocking the potential pass with the orientation of the body and the raised hand on that side. The other side, in contrast, was not covered and offered itself for a pass (cf. Figure 1). The stimuli were projected in front of the participants with a projector (Optoma X320) at the screen wall (height: 140 cm, width: 200 cm).

On the first day of practice, participants were shown four short videos of a professional basketball player performing a pass with or without a head fake to the left or the right side to familiarize themselves with the to-be-executed movements. Afterwards, the participants were given a basketball and were instructed to place it onto a button on the desk in front of them. In the following, this position will be referred to as the starting position. The participants were also given a black cap with a white stripe in the middle of the visor (posterior to anterior). This cap, in combination with a camera mounted above the participants’ starting position, allowed the analysis of the head movement for each trial. Then, participants were instructed to perform a passing action to the side that was not defended by the basketball player presented. The pass should be carried out with or without a head fake, depending on the color of the basketball player’s shirt. For both passes with and without head fakes, the participants were instructed to initiate the head movement and the movement of the basketball simultaneously. The assignment of the passes with or without head fake to the corresponding shirt colors was counterbalanced between the participants. The participants were instructed to execute the pass only after an auditory GO-signal (300Hz, jigsaw soundwave) was given and only to initiate the passing action after they planned their reaction. This GO-signal was either presented simultaneously with the visual cue (ISI 0 ms) or 400 ms, 800 ms or 1200 ms after the stimulus onset.

**Fig. 2:**
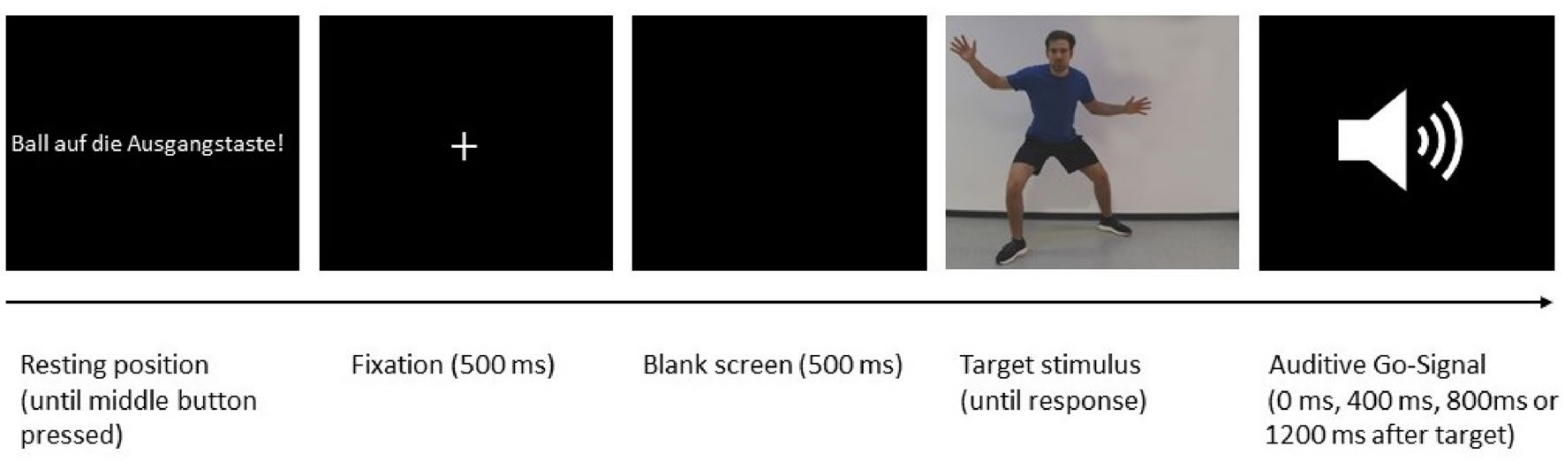
Trial sequence. Each trial started with the instruction to place the basketball on the start button (“Ball on start button”). A white fixation cross appeared, which was followed by a blank screen. Afterwards, the target stimulus of the basketball player was displayed, until the participants gave their response after the auditory GO-signal. The auditive cue was played either directly with the display of the target (0 ms) or with a short delay (400 ms, 800 ms, or 1200 ms). The participants responded by either performing a pass with or without head fake to the left or right and pressing the corresponding response button with the basketball. In this example, participants would have to perform a pass (with or without head fake, depending on the assignment of the type of pass to the shirt color) to the right “open” side after the auditive cue was played

The trials started with an instruction presented on the screen, asking the participant to place the basketball on the start button (starting position), which started the individual trial. First, a white fixation cross appeared for 500 ms in the middle of the screen. Afterwards a blank screen was presented for 500 ms before the target stimulus of the basketball player was displayed. The target stimulus remained on the screen until participants pressed one of the response buttons with the basketball (on the left or right side of the basketball apparatus). When the participants responded before the auditory GO-signal was given, the German words “Zu schnell” (too fast) were shown for 1000 ms. After participants’ passing movement to the left/right, the instruction to place the basketball on the start button was again displayed and the next trial started. The trial sequence is illustrated in Fig. 2.

On the first day of practice, participants began the experiment with a practice block of 32 trials in a fixed order and received feedback on their pass direction and head movement after each trial by the instructor. If a participant struggled with performing the correct head turning and passing movement even at the end of the practice block (4 or more wrong answers in the last 8 trials), the participants had to complete the whole practice block again. After the practice block was completed, the participants performed 4 training blocks of 80 trials each. The trials varied with regard to *type of pass* (pass with head fake vs. pass without head fake) and *ISI* (0 ms, 400 ms, 800 ms, 1200 ms), and were presented in randomized order. Thus, each condition was repeated ten times within a block. The direction in which the pass had to be performed (i.e., left vs. right) was equally distributed and not manipulated as an experimental factor. On the second through fifth day of training, participants only performed a short practice block of 8 trials in a fixed order before the start of the 4 training blocks of 80 trials.

### Data analysis

We analyzed initiation times (ITs), movement times (MTs), error rates (ERs) as well as the coefficient of variation of the IT (IT_cv_) as dependent variables. Initiation time was the time interval between presentation of the GO-signal and the point in time when the participant lifted the ball from the starting position. The movement time started with the end of the initiation time, and it was terminated when the participant pressed the ball against the response button. Trials were excluded if either the initiation time or movement time deviated more than three standard deviations from the cell mean, calculated separately for each participant, *ISI*, *type of pass*, and *day of practice* (1.6%). Furthermore, all incorrect movements were excluded from further IT and MT analyses, that is, movements which were initiated before the GO-Signal (0.3%), movements to the wrong response button (0.2%), movements with a head movement to the wrong side (2.2%), and movements with no or a delayed head response (0.9%). We also analyzed error rates, that is, movements to the wrong response buzzer and/or passing movements with a head movement to the wrong side (3.7%). The coefficient of variation of the IT (IT_cv_), as an indicator of the variability in the reactions, was calculated as the standard deviation in the response initiation time (SD_IT_) divided by the mean of the initiation time (M_IT_), multiplied by 100 (Flehming et al., 2007).

Before performing the statistical analysis, the normal distribution was checked with the Shapiro–Wilk test for all analyzed data and homoscedasticity with Levene’s test. All dependent variables were analyzed with repeated measures ANOVAs with the factors *type of pass* (pass with head fake, pass without head fake), *ISI* (0 ms, 400 ms, 800 ms, 1200 ms), and *day of practice* (Day 1, Day 2, Day 3, Day 4, Day 5) and significant interactions of the factors were analyzed with paired t-tests, in which *p*-values were all pairwise post-hoc corrected (adjusted to Holm-Bonferroni; Holm, 1979). The alpha-level chosen for significance was 0.05. A violation of the sphericity-assumption resulted in a correction of the *p*-values according to Greenhouse–Geisser. Partial eta-squared (*ɳ*_*p*_^*2*^*)* values of 0.01, 0.06, and 0.14 were interpreted to indicate small, medium, or large effects, respectively (Richardson, 2011). Epsilon (*ε*) represents the degree to which sphericity was present, with lower values indicating a greater violation of sphericity.

## Results

### Initiation times

An ANOVA with mean initiation time as dependent variable and *type of pass* (pass without head fake vs. pass with head fake), *day of practice* (days 1, 2, 3, 4 and 5), and *ISI* (0 ms, 400 ms, 800 ms, 1200 ms) as repeated measures revealed a main effect for the factor *type of pass*: initiation times were slower for passes with a head fake (*M* = 444 ms) than for passes without a head fake (*M* = 432 ms), *F*(1, 23) = 8.28; *p* = 0.009; *ɳ*_*p*_^*2*^ = 0.265. Also, the ANOVA indicated a main effect of the factor *ISI*, revealing a reduction of initiation times with increasing *ISI*, *F*(1.374, 31.608) = 597.08; *p* < 0.001; *ɳ*_*p*_^*2*^ = 0.963; *ε* = 0.458, from ISI 0 ms (*M* = 622 ms) over ISI 400 ms (*M* = 392 ms) over ISI 800 ms (*M* = 370 ms) to ISI 1200 ms (*M* = 367 ms). There was also a main effect for the factor *day of practice*: a general, but not monotonous, decrease in the initiation times of the participants from Day 1 (469 ms) to Day 2 (427 ms), to Day 3 (439 ms), to Day 4 (430 ms), and to Day 5 (423 ms), *F*(1.428, 32.846) = 4.84; *p* = 0.023; *ɳ*_*p*_^*2*^ = 0.174; *ε* = 0.357. The ANOVA also revealed an interaction of *ISI* with *type of pass, F*(1.639, 37.693) = 35.95; *p* < 0.001; *ɳ*_*p*_^*2*^ = 0.610*; ε* = 0.546, as well as an interaction of *ISI* with *day of practice, F*(4.032, 92.731) = 8.10; *p* < 0.001; *ɳ*_*p*_^*2*^ = 0.261*; ε* = 0.336, and of *day of practice* with *type of pass, F*(1.462, 32.788) = 5.51; *p* = 0.016; *ɳ*_*p*_^*2*^ = 0.193*; ε* = 0.356. The ANOVA indicated a three-way interaction between *type of pass*, *ISI,* and *day of practice F*(5.473, 125.890) = 5.47; *p* = 0.012; *ɳ*_*p*_^*2*^ = 0.115*; ε* = 0.456 (Table 1).

**Table 1:** Fake Production costs in the initiation times

	Day 1	Day 2	Day 3	Day 4	Day 5
ISI 0 ms	57 ms	46 ms	30 ms	30 ms	23 ms
ISI 400 ms	42 ms	11 ms*	7 ms*	5 ms*	7 ms*
ISI 800 ms	1 ms	− 5 ms	− 3 ms	− 8 ms	− 7 ms
ISI 1200 ms	9 ms	− 3 ms	− 6 ms	− 2 ms	−4 ms

Mean fake production costs expressed as difference between passes with head fakes and passes without head fakes in the initiation times depicting the triple interaction of *type of pass, day of practice* and *ISI*. Asterisks indicate significant differences in the initiation times of the respective Day compared to Day 1 of the same ISI (*p* < .05)

To evaluate the differences in initiation times between passes with and without head fakes for the different ISIs over the course of practice (i.e., the potential decrease of fake-production costs with practice in dependence of the ISI), single comparisons (paired t-tests adjusted to Holm-Bonferroni; Holm, 1979) were conducted. At the ISI of 0 ms, participants responded faster for passes without than for passes with head fakes on Day 1 of practice (639 ms vs. 697 ms, *t*(23) = − 4.08, *p* < 0.001, *d* = 0.83), Day 2 (595 ms vs. 641 ms, *t*(23) = − 5.89, *p* < 0.001, *d* = 1.20), Day 3 (606 ms vs. 636 ms, *t*(23) = − 5.20, *p* < 0.001, *d* = 1.06), Day 4 (592 ms vs. 622 ms, *t*(23) = − 5.27, *p* < 0.001, *d* = 1.07), and Day 5 (586 ms vs. 609 ms, *t*(23) = − 6.92, *p* < 0.001, *d* = 1.41). At the ISI of 400 ms, participants showed faster initiation times for passes without than for passes with head fakes on Day 1 of practice (399 ms vs. 420 ms, *t*(23) = − 3.57, *p* = 0.012, *d* = 0.73), but not on the following days (all *ps* > 0.05). There were no significant differences in the initiation times between direct passes and head fakes at the ISI of 800 ms or 1200 ms on all days of practice (all *ps* > 0.05).

As the previous analysis revealed significant fake production costs for ISIs of 0 ms (for all 5 days) and 400 ms (on Day 1), we used paired samples t-tests to investigate if the fake production costs for the short ISIs were reduced from Day 1 to Day 5, as was hypothesized. Paired t-tests revealed a descriptive, but not significant, reduction of the fake production costs at the ISI of 0 ms from Day 1 (57 ms) to Day 2 (46 ms), Day 1 to Day 3 (30 ms), Day 1 to Day 4 (30 ms), and Day 1 to Day 5 (23 ms) (all *ps* > 0.05). At the ISI 400 ms, the paired t-test revealed a significant reduction of the fake production costs from Day 1 of practice to Day 2 (42 ms vs. 11 ms, *t*(23) = 3.11, *p* = 0.012, *d* = 0.63).

**Fig. 3:**
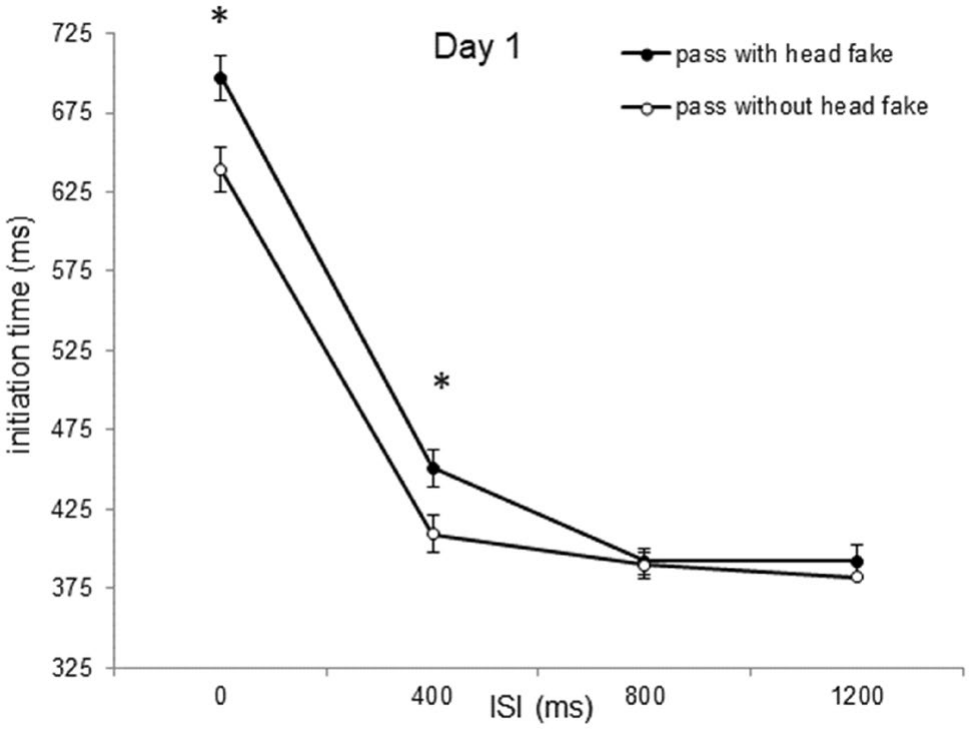
Initiation times of Day 1. Initiation times of day 1 as a function of *type of pass* and *ISI* (error bars show standard errors of the mean difference of passes with and without head fakes per ISI). Asterisks indicate significant effects (*p* <0 .05)

**Fig. 4:**
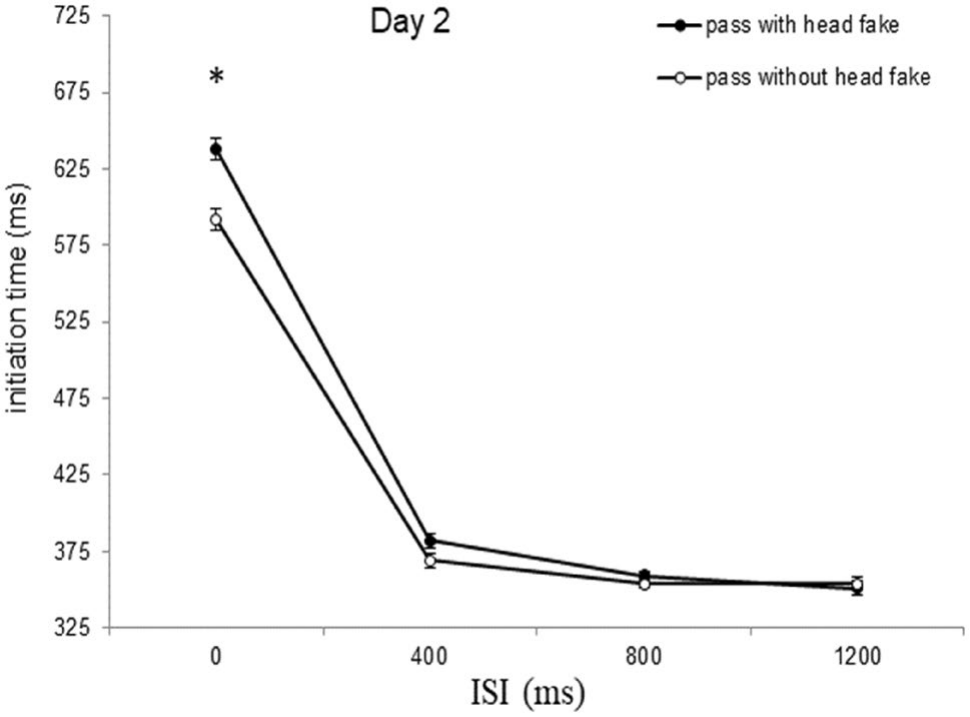
Initiation times of Day 2. Initiation times of day 2 as a function of *type of pass* and *ISI* (error bars show standard errors of the mean difference of passes with and without head fakes per ISI). Asterisks indicate significant effects (*p* <  0.05)

Together, head-fake production costs only occurred at the ISI of 0 ms on all five days and at the ISI of 400 ms on the first day of practice. The figures for the mean initiation times for Day 1 (Fig. 3) and Day 2 (Fig. 4) are shown below.

### ITcv

**Fig. 5:**
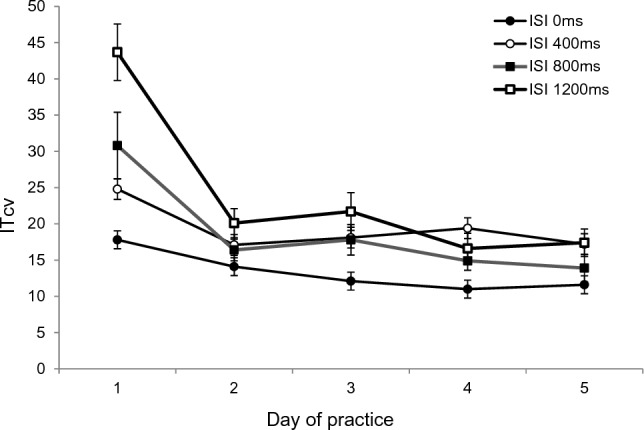
IT_cv_ for all ISI from Day 1 to Day 5 of practice. IT_cv_ as a function of *day of practice* and *ISI* (error bars show standard errors of passes with and without head fakes per ISI)

The ANOVA with the mean variation coefficient of initiation times (IT_cv_) as dependent variable and *type of pass* (pass without head fake vs. pass with head fake), *day of practice* (days 1, 2, 3, 4 and 5), and *ISI* (0 ms, 400 ms, 800 ms, 1200 ms) as repeated measures revealed a main effect for the factor *type of pass:* IT_cv_ was higher for passes with head fakes (19.9) than for passes without head fakes (17.8), *F*(1.23) = 4.35; *p* = *0.0*41; *ɳ*_*p*_^*2*^ = 0.16. The ANOVA also indicated a main effect for the factor *ISI*: IT_cv_ generally (but not monotonously) increased with increasing ISI from ISI 0 ms (13.3) to ISI 400 ms (19.3), to ISI 800 ms (18.8), to ISI 1200 ms (23.9), *F*(1.643, 39.429) = 11.78; *p* < *0.0*01; *ɳ*_*p*_^*2*^ = 0.34, as well as a main effect for the factor *day of practice*, as shown by a decrease of the IT_cv_, even though not monotonous, from Day 1 (29.3) to Day 2 (16.9), to Day 3 (17.4), to Day 4 (15.5), to Day 5 (15.1), *F*(4, 92) = 23.96; *p* < *0.0*01; *ɳ*_*p*_^*2*^ = 0.51. Similar to the results of the reaction times, the greatest changes could be seen after the first day of practice. Also, the ANOVA revealed a two-way interaction of the factors *ISI* and *day of practice*, *F*(12, 276) = 3.55; *p* = *0.0*22; *ɳ*_*p*_^*2*^ = 0.13 (cf. Figure 5) which, however, was not the focus of the current study and thus, is not further evaluated here. None of the other two-way interactions and neither the three-way interaction reached significance (all *p*s > 0.05).

### Movement times

**Fig. 6:**
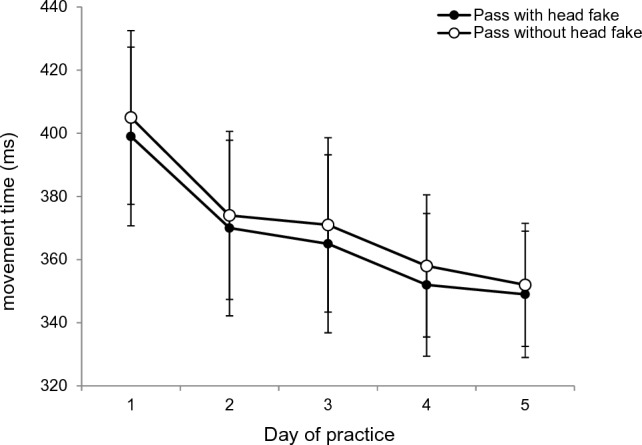
Movement times for passes with and without head fakes from Day 1 to Day 5. Mean movement times of passes with and passes without head fakes as a function of *day of practice* (Error bars show standard errors of passes with and without head fakes per day)

An ANOVA with mean movement times as dependent variable and *type of pass* (pass without head fake vs. pass with head fake), *day of practice* (days 1, 2, 3, 4 and 5) and *ISI* (0 ms, 400 ms, 800 ms, 1200 ms) as repeated measures revealed slightly faster movement times for passes with a head fake (*M* = 367 ms) than for passes without a head fake (*M* = 372 ms), *F*(1, 23) = 5.58; *p* = *0.0*27; *ɳ*_*p*_^*2*^ = 0.19. Also, the ANOVA indicated a main effect for *day of practice*, *F*(1.982, 45.587) = 5.58; *p* = 0.007; *ɳ*_*p*_^*2*^ = 0.19; *ε* = 0.496, as participants showed a reduction of the mean movement times from Day 1 (*M* = 402 ms), to Day 2 (*M* = 372 ms), to Day 3 (*M* = 368 ms), to Day 4 (*M* = 355 ms), to Day 5 (*M* = 351 ms) (cf. Figure 6). The main effect for the factor *ISI* was also significant, *F*(1.657, 38.102) = 4.05; *p* = *0.0*32; *ɳ*_*p*_^*2*^ = 0.15; *ε* = 0.552, showing a slight, but not monotonous, increase in MTs from ISI 0 ms (*M* = 366 ms) to ISI 400 ms (*M* = 373 ms), ISI 800 ms (*M* = 371 ms), and ISI 1200 ms (*M* = 369 ms). None of the interactions reached significance (all *ps* > 0.05).

*Post-hoc* t-tests revealed significantly shorter mean movement times at the ISI of 0 ms (366 ms) than at the ISI of 400 ms (373 ms), *t*(23) = − 3.367, *p* = 0.009, *d* = 0.68. The comparisons with the other ISIs did not reach significance (all *ps* > 0.05).

### Error rates

The ANOVA with the mean error rate (in %) as dependent variable and *type of pass* (pass without head fake vs. pass with head fake), *day of practice* (days 1, 2, 3, 4 and 5), and *ISI* (0 ms, 400 ms, 800 ms, 1200 ms) as repeated measures revealed higher error rates for passes with a head fake (*M* = 5.3%) than for passes without a head fake (*M* = 2.0%), *F*(1, 23) = 49.21; *p* < 0.001; *ɳ*_*p*_^*2*^ = 0.68. Also, the ANOVA indicated higher error rates for the ISI of 0 ms (*M* = 7.6%) than for ISI 400 ms (*M* = 2.4%), ISI 800 ms (*M* = 2.1%), and ISI 1200 ms (*M* = 2.6%), *F*(1.263, 29.045) = 34.53; *p* < 0.001; *ɳ*_*p*_^*2*^ = 0.60; *ε* = 0.421. The ANOVA also revealed a consistent reduction of the mean error rate from Day 1 (7.6%), to Day 2 (4.4%), to Day 3 (2.6%), to Day 4 (2.1%), to Day 5 (1.6%), *F*(1.929, 44.374) = 17.12; *p* < 0.001; *ɳ*_*p*_^*2*^ = 0.42; *ε* = 0.482. The analysis also indicated an interaction of *ISI* with *day of practice, F*(3.827, 88.023) = 3.97; *p* = 0.006; *ɳ*_*p*_^*2*^ = 0.14; *ε* = 0.319, an interaction of *ISI* with *type of pass*, *F*(1.417, 32.592) = 35.034; *p* < 0.001; *ɳ*_*p*_^*2*^ = 0.60; *ε* = 0.333, and an interaction of *type of pass* with *day of practice, F*(1.559, 36.772) = 16.921; *p* < 0.001; *ɳ*_*p*_^*2*^ = 0.42; *ε* = 0.400. The ANOVA revealed a three-way interaction between *ISI*, *type of pass,* and *day of practice*, *F* (5.934, 136.474) = 4.16; *p* < 0.001; *ɳ*_*p*_^*2*^ = 0.15; *ε* = 0.494, indicating a reduction of the error rates with increasing ISI duration and increasing practice of the participants, with higher mean error rates for passes with than for passes without head fakes (Table 2).

**Table 2:** Fake Production Costs in the Error Rates

	Day 1 (%)	Day 2 (%)	Day 3 (%)	Day 4 (%)	Day 5 (%)
ISI 0 ms	12.8	11.6	6.5*	6.6*	3.2*
ISI 400 ms	8.7	0.5*	1.2*	0.7*	0.8*
ISI 800 ms	3.2	1.0	0.3	0.1	0.4
ISI 1200 ms	5.2	0.3	1.4	0.0	0.4

Mean fake production costs expressed as difference between passes with head fakes and passes without head fakes in the error rate depicting the triple interaction of *type of pass, day of practice* and *ISI*. Asterisks indicate significant differences in the error rate of the respective Day compared to Day 1 of the same ISI (*p* < .05)

**Fig. 7:**
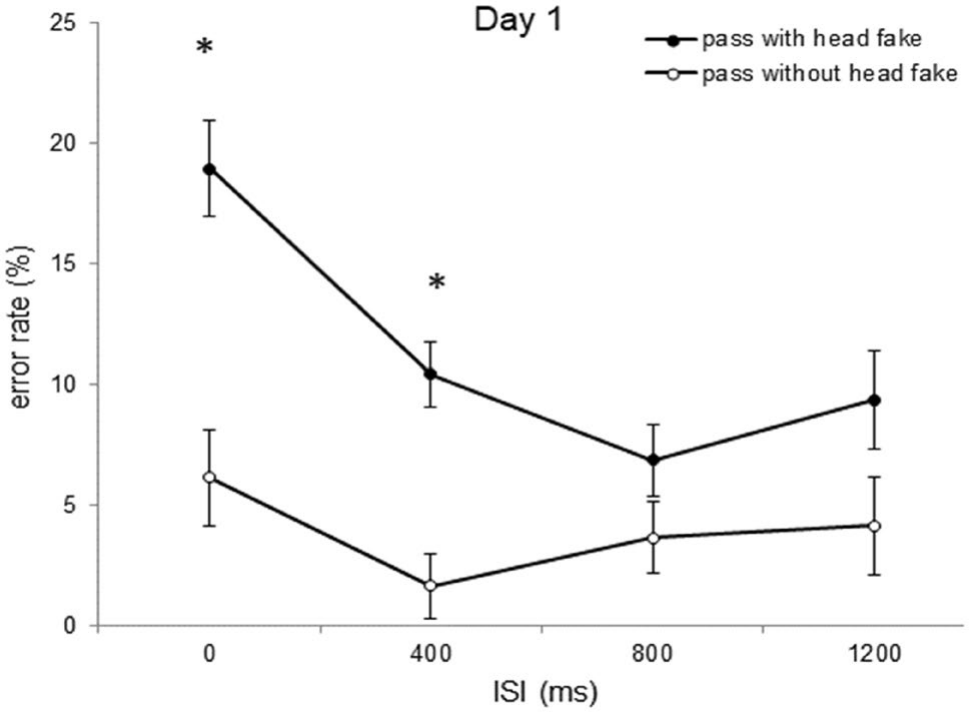
Error rate of Day 1. Error rates as a function of *type of pass* and *ISI* for day 1 of practice (error bars show standard errors of the mean differences of fakes and direct passes per ISI). Asterisks indicate significant effects (*p*  < 0.05)

**Fig. 8:**
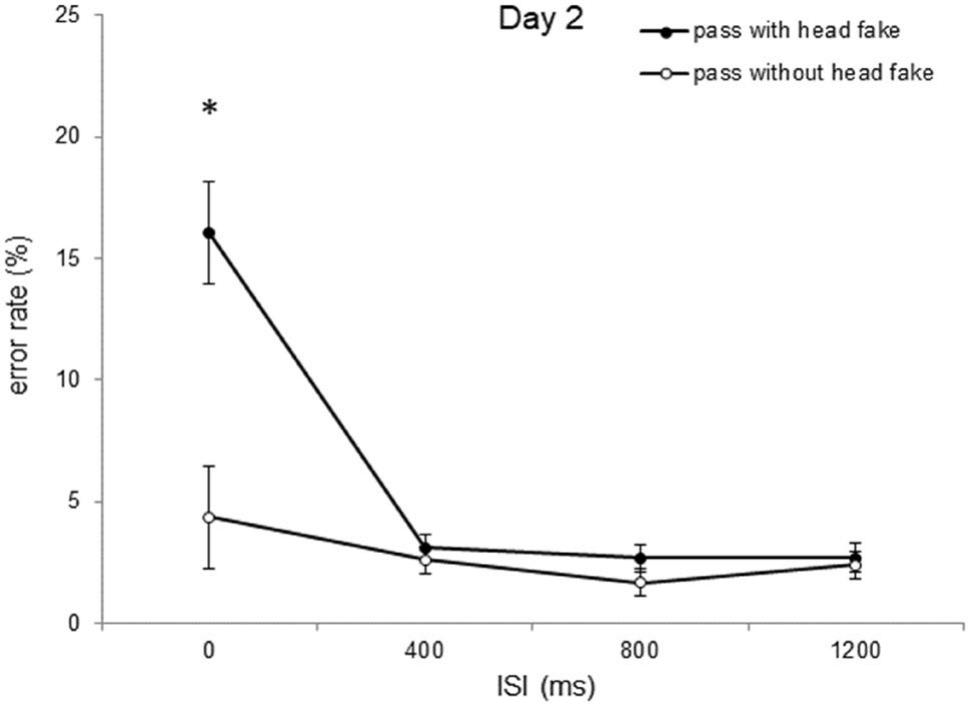
Error rate of Day 2. Error rates as a function of *type of pass* and *ISI* for day 2 of practice (error bars show standard errors of the mean differences of fakes and direct passes per ISI). Asterisks indicate significant effects (*p* < 0.05)

To evaluate the development of the head-fake production costs for the different ISIs over the course of practice (i.e., from Day 1 to Day 5), single comparisonswere conducted. For the ISI of 0 ms, participants performed significantly less errors for passes without head fakes than for passes with head fakes on Day 1 of practice (6.1% vs. 18.9%, *t*(23) = -6.41, *p* < 0.001, *d* = 1.31), Day 2 (4.3% vs. 16.0%, *t*(23) = -5.55, *p* < 0.001, *d* = 1.13), Day 3 (2.2% vs. 8.8%, *t*(23) = − 6.11, *p* < 0.001, *d* = 1.17) and Day 4 (2.2% vs. 8.9%, *t*(23) = -5.83, *p* < 0.001, *d* = 1.19), but not on Day 5 (2.5% vs. 5.7%, *t*(23) = -2.61, *p* > 0.05, *d* = 0.53). At the ISI of 400 ms, participants showed lower error rates for passes without head fakes than for passes with head fakes on Day 1 of practice (1.6% vs. 10.4%, *t*(23) = -6.54, *p* < 0.001, *d* = 1.33), but not on the following days (all *ps* > 0.05). There were no significant differences in the error rates between passes with and without head fakes at the ISI of 800 ms or 1200 ms on all days of practice (all *ps* > 0.05). The mean percentages of error rates as a function of *type of pass* and *ISI* of Day 1 (Fig. 7) and Day 2 (Fig. 8) of practice are shown below.

Paired t-tests revealed a reduction of the fake production costs in the error rates at the ISI of 0 ms from Day 1 (12.8%) to Day 3 (6.5%), *t*(23) = 3.02, *p* = 0.018, *d* = 0.61, Day 1 to Day 4 (6.6%), *t*(23) = 2.99, *p* = 0.018, *d* = 0.61, and Day 1 to Day 5 (3.2%), *t*(23) = 4.51, *p* < 0.001, *d* = 0.92, but not from the first day of practice to the second day (*p* > 0.05). Another paired t-test indicated a significant reduction of the fake production costs in the error rates at the ISI of 400 ms from Day 1 (8.7%) to Day 2 (0.5%), *t*(23) = 6.38, *p* < 0.001, *d* = 1.30, from Day 1 to Day 3 (1.3%), *t*(23) = 4.64, *p* < 0.001, *d* = 0.95, Day 1 to Day 4 (0.7%), *t*(23) = 5.41, *p* < 0.001, *d* = 1.11, and Day 1 to Day 5 (0.8%), *t*(23) = 5.24, *p* < 0.001, *d* = 1.07.

## Discussion

The present study aimed to investigate the effects of extensive practice with passing movement execution for passes with and without head fakes in a basketball setting on the fake production costs for basketball novices. Participants practiced the task on 5 consecutive days. Similar to previous studies (Güldenpenning et al., 2023; Kunde et al., 2019; Wood et al., 2017), significant fake production costs were observed when participants had no (ISI = 0 ms) or only limited time (ISI = 400 ms) to prepare the fake action. The results from the first day of practice fit well with the ones from the previous study (Güldenpenning et al., 2023), with similar initiation times and error rates, which have been reduced by increases of the ISI duration. Expanding on previous research, these results indicate that practicing a head fake reduces its performance costs. Moreover, performing passes with and without head fakes generally stabilizes participants performance over the course of practice.

The fake production costs found here are suggested to be caused by response-response incompatibility costs (Hazeltine, 2005; Heuer, 1995; Peterson, 1965), as performing a head fake requires the execution of two spatially incompatible movements, namely the head turn (e.g., to the left) and the passing movement (e.g., to the right). The present study shows that these fake production costs can be reduced by two factors. First, they can be reduced when participants are given enough time to prepare the movement, which is evident in decreasing fake production costs for increasing ISIs. Second, fake production costs can be reduced through practice, which can be seen in decreasing fake production costs for the ISI of 0 ms. However, the decrease in fake-production costs was only significant in ERs but not in ITs. Regarding the ISI of 400 ms, fake production costs were eliminated after only one day of practice. It was predicted that extensive physical practice of producing head fakes would strengthen stimulus–response associations between the action-specifying stimulus (i.e., the presented basketball player) and the to-be-executed action (i.e., a pass with or without head fake, to the left or right side), which might result in an automatic translation of a stimulus into a response (Hommel, 2000). If so, there should be no fake-production costs at the ISI of 0 ms anymore, as the action selection process (which is the origin of the production costs of a head fake; Güldenpenning et al., 2023) is skipped because the response is already uniquely specified by the stimulus. This was not the case, as the fake production costs in ITs were not eliminated through practice. As, however, fake-production costs were eliminated through practice for the ISI of 400 ms, it is argued here that practice nevertheless strengthened the stimulus–response associations. Based on these findings, one can assume that the response selection time dropped below 400 ms due to practice.

Regarding the effects of practice, both for the general decrease in ITs and for the decrease of the fake production costs, these were most pronounced from Day 1 to Day 2, indicating that one day of practice is enough to substantially improve the reaction times in passes with and without head fakes. This interpretation is supported by the analysis of the IT_cv_, which is not affected by test repetition effects (Flehmig et al., 2007). Practice, thus, stabilized performance of playing passes with and without head fakes in basketball over the course of practice. However, more practice would have been necessary to further reduce fake-production costs for the ISI of 0 ms. Previous studies using cueing paradigms showed that participants still improved their performance after 20 days of practice (Sudevan & Taylor, 1987). Future studies should therefore investigate longer and more extensive practice periods to evaluate whether participants could completely eliminate the productions costs of head fakes. This would be important for sports practice as an increase of the initiation time of an action of 100 ms increased the chance that the opponent would expect a deceptive movement by 10% (Kunde et al., 2019). Performing a deceptive movement without fake production costs would therefore reduce the chance that the defending player expects a fake movement and adapts the response.

The present study comes with some limitations, however, that concern the transferability into practice. First, participants did not decide themselves when to use the deceptive action but were merely instructed when to perform which action depending on one specific stimulus. There are indications, that there could be differences in fake-production costs between situations where the participants could decide themselves which action to perform in comparison to simple reactions to visual information (i.e., reacting to a single defensive player already covering one side), as these actions seem to have fundamentally different mechanisms (Weller et al., 2018). Future research should examine fake-production costs in situations where the attacking player themselves decides whether to play a pass with or without a head fake. Second, the participants in this study were basketball novices. So even though this study could show that extensive practice with passing movement execution for passes with and without head fakes can reduce the fake production costs, this may only apply to unexperienced basketball players. Because this paradigm is fairly new and research on fake production costs in sports is still rare, we can only speculate whether basketball experts will also show fake production costs. It is well known that practice strengthens stimulus–response associations, even up to a level of automaticity (Hommel, 2000). In a real sports context, for example, a basketball expert might automatically activate a specific action (e.g., a specific technical maneuver) in response to a perceived trigger (e.g., a specific tactical constellation). Such an automatic stimulus–response association implies that responses as such do not need to be selected anymore, as it is disposed to the stimulus. Accordingly, basketball experts might show no fake-production costs if they perform a head fake in a highly trained situation. This needs to be addressed in future studies. Another limiting factor was that participants performed the task alone and not in an interaction scenario with a defending player. This could have reduced the production costs of the passes with head fakes as Kunde et al. (2019) could show additional cognitive costs could be observed in an interaction scenario compared to a situation without a competitor. Such additional costs that may arise when executing a deceptive action with an interaction partner could be caused by rule violation (Foerster et al., 2017) or the mental imagining of the opponent’s reaction (Kunde et al., 2018). Lastly, only the effects of 5 days of practice were studied here. As discussed above, the participants still improved their general reaction times at Day 5 of practice and showed a further reduction of the fake production costs at the ISI of 0 ms. Therefore, it is possible that prolonged practice could have eliminated the production costs of the passes with head fakes even when the participants did not have time to mentally prepare their action.

## Conclusion

First, our study provides further evidence that fake production costs occur during response selection and are suggested to be caused by response-response incompatibility costs (Diedrichsen et al., 2001). Not only are the initiation times for passes with head fakes increased but the participants also made more errors executing these actions when they had no or little time to prepare their reaction (i.e., ISI 0 ms and 400 ms) compared to passes without head fakes. Second, practicing the pass with head fake reduced fake production costs, indicating that expertise with the deceptive movement could be essential to reach the maximal potential of the head fake as increased initiation times might increase the opponent’s anticipation of a deceptive action (cf. Kunde et al., 2019). Third, even extensive practice might not be able to completely eliminate the fake production when participants have no time to select a pass with head fake in advance. Basketball players should therefore try to mentally prepare their actions in advance e.g., while approaching the defending player. This is not only advantageous when performing deceptive movement but also for passes without head fakes, as initiation times and error rates still decreased with an increasing ISI.

Future studies should investigate whether sports specific experts also show fake production costs when performing deceptive actions. Basketball players, who are familiar with the movement, could possibly have enough expertise with the movement planning to skip the process of response selection, which could eliminate fake production costs. It is also possible that expert players might use other strategies to perform passes with head fakes, for example, delaying the movement programming into the phase of movement execution (deferred programming hypothesis; Spijkers et al., 1997), in order to prevent increased initiation times as opponents are more likely to suspect the deceptive intent of the attacker (cf. Kunde et al., 2019). Further research into the effectiveness of different types of shot fakes in basketball and how they might be affected by fake production costs is also important, as a recent study by Meyer et al., (2022b) suggested that high shot fakes are even more effective in deceiving a defending player than head fakes. But it is yet unknown whether attackers show a form of fake production costs when using a high shot fake compared to a direct shot. Last, it seems worthwhile for future research to focus on interaction scenarios, which allow the measurement of additional cognitive and social costs when performing deceptive movements.

## Data Availability

The datasets generated during and analyzed during the current study are available from the corresponding author on reasonable request.
